# Pathology

**Published:** 1907-11

**Authors:** R. L. Anderson

**Affiliations:** Plant City, Fla.


### PATHOLOGY.

By R. L. Anderson, D. D. S., Plant City, Fla.

As we all know, Dental Pathology embraces every diseased and abnormal condition of the mouth, and we can not very well study or put into practice Pathology, without a fair knowledge of Bacteriology and Etiology, as the three are so closely connected.

When we stop to study the nature of a disease, we cannot help wondering what caused it, which is almost invariably bacterial infection, and in order to accomplish a cure or have any degree of success we must administer something that will eradicate or destroy the action of those germs.

I am afraid it is too often the case that many of us neglect doing our duty to our patients in the treatment and study of diseases, other than defects of the teeth.

Very often we allow our patients to leave our office with a bad case of Pyorrhea or Riggs Disease or some other serious disease of the gums; when it is just as necessary that they should be cared for as the teeth, for we see persons almost every day losing perfectly sound teeth which could have been saved had their gums been properly cared for.

We do not impress this fact upon our patients as we should, the necessity of having their teeth cleaned and their gums looked after regularly.

So many times we shirk our duty in this respect, rather than butt up against trouble. I noticed that among the boys in college, there were but few of them willing to take a case of Pyorrhea, or any other disease of the gums; they were looking for something that the could turn off in a hurry with as little work as possible.

I remember one day, an old man came into the Infirmary with his head twice the size of an ordinary man's head and when the boys saw him they began at once to leave. The demonstrator began to look for some one to take the case but everyone he asked, declined "with thanks." He was finally forced to care for the old man himself. Those of us who witnessed the diagnosis of the case, learned that it was a typical case of Mercurial Stomatitis.

It is very necessary that boys in college take advantage of every opportunity to treat these diseases in order to be able to battle against them in after practice.

I am afraid that too many of us seek an easy living out of Dentistry; it seems that all many dentists are after, is the Almighty Dollar, and they do not care a straw what becomes of their patients after they leave their office. I find that if we do our duty to our patients, as we should, dentistry is no easy job, but is one of the toughest propositions I ever butted up against. I have done some pretty hard work; even stood in a ditch up to my knees shoveling mud, day after day, and doubtless by the time I have finished reading this paper you will think that I should be there now; but I am here for the good of humanity and just as soon as I get to where I do not do my duty to the best of my ability, I hope some one will run me back to the good old farm in the old "Tar-heel" State.

Gentlemen, if you will pardon me, I will mention a case or two in my practice:

A countryman about thirty-five years of age come to me last summer and said, "Dr. Bryan, Dr. C——— told me to to come to you and get this loose tooth pulled." I examined his mouth and found it in as bad condition as I ever saw one. The crowns of all the teeth were covered with tartar, and pus was oozing from around the roots of all the teeth, the gums having receded half way to the apex, leaving many of the teeth very loose; one of which was the lower incisor that he had me extract. I also extracted an upper first bicuspid root which had been the cause of a chronic abscess for fcur years and just above the orifice of the fistulous opening there was a growth about half the size of a quail's egg; I washed out the fistula with peroxide of hydrogen and burned it out well with carbolic acid. He stated that Dr. C——— had been treating him three months for catarrh of the head; I told him I did not wonder at him having catarrh with his mouth in that condition and advised him to have his teeth and gums treated and I thought it would cure his catarrh.

After giving him this advice I made an engagement with him and he paid me in advance for fear he would spend his money and not get his work done. He came back on time and I spent two hours and a half cleaning his teeth, then gave him a prescription for an antiseptic and astringent mouth wash and told him to let me know how he was getting along. About three months later he walked up to me and spoke, and upon my word, I did not know the man as he had improved so much. I asked him how his catarrh was, he said that he could not tell that he had ever had catarrh and that he felt better than he had for ten years. I examined his gums and found them to be in a healthy condition and very little sign of the tumor.

This case proves to us the good we can accomplish for our patients by giving the proper advice; it is not only beneficial to them but to us as well. This man will never fail to speak well of me every time an opportunity presents itself, and you have no idea how many persons will come to our offices through the influence of such persons.

I would like to mention another case I treated. A very prominent man of my town came to me with a blind abscess on one of his upper central incisors. I opened it and treated it in the usual way and every time I would put a treatment in it and seal it, he would come back in a few minutes with it giving him trouble. I worried with it for at least two months and never did get it so I could seal it with a treatment in it.

After worrying with it for the above mentioned period, I ordered a bottle of Pustolene having seen it advertised very highly for cases of this kind; I thought this was a case to test its virtue. I dried out the root canal well, added enough Creosote to the Pustolene to make a soft paste, then pumped this with a smooth broach, pumping a small bit through the apex, then rolled cotton around the broach, saturated it well with Pustolene and inserted it in the root and sealed it up. It has been in there now for three months and although it does not give him any trouble he says that it does not feel very pleasant.

Another gentleman presented himself to me about this time also, with a blind abscess on an upper lateral. I thought to myself, I will try Pustolene on you and see what it will do for you. He said that he had been troubled with it for ten days. I opened it ,and cleaned it out as best I could with a drill and broach, dried it out well and treated it just as I did the above mentioned case. I expected him back the next day with a blooming abscess sure enough, but it was several days before he came, and said it gave him trouble three days but was easy then. The same treatment has been in there now for two months and has never given him any more trouble.

One other thought and I am done. I want to call your attention to the fact that physicians receive a good deal of our practice through the ignorance of the people. They do not seem to realize that Dentists make a special study of all diseases of the mouth, and that dentistry is a specialty of medicine. Whenever they have a disease of the mouth, or have trouble with their jaws after having teeth extracted; they go straight for the physician. We should do something to overcome this and the best way I can think of is to render to every patient the best possible service and let them know that we are something more than mere "tooth pullers" and "tooth pluggers." If we will do this, we will not only add dollars and cents to our pockets, but will be appreciated more by the public.

"Then let us be up and doing, with a heart for any fate,
Still achieving, still pursuing, learn to labor and to wait."

